# An Online Assessment to Evaluate the Role of Cognitive Biases and Emotion Regulation Strategies for Mental Health During the COVID-19 Lockdown of 2020: Structural Equation Modeling Study

**DOI:** 10.2196/30961

**Published:** 2021-11-02

**Authors:** Ivan Blanco, Teresa Boemo, Alvaro Sanchez-Lopez

**Affiliations:** 1 Department of Clinical Psychology School of Psychology Complutense University of Madrid Pozuelo de Alarcon Spain; 2 Cardenal Cisneros University Centre Alcala de Henares Spain

**Keywords:** COVID-19, emotion regulation, cognitive biases, psychological adjustment, resilience

## Abstract

**Background:**

Extant research supports causal roles of cognitive biases in stress regulation under experimental conditions. However, their contribution to psychological adjustment in the face of ecological major stressors has been largely unstudied.

**Objective:**

We developed a novel online method for the ecological examination of attention and interpretation biases during major stress (ie, the COVID-19 lockdown in March/April 2020) and tested their relations with the use of emotion regulation strategies (ie, reappraisal and rumination) to account for individual differences in psychological adjustment to major COVID-19–related stressors (ie, low depression and anxiety, and high well-being and resilience).

**Methods:**

Participants completed an online protocol evaluating the psychological impact of COVID-19–related stressors and the use of emotion regulation strategies in response to them, during the initial weeks of the lockdown of March/April 2020. They also completed a new online cognitive task designed to remotely assess attention and interpretation biases for negative information. The psychometric properties of the online cognitive bias assessments were very good, supporting their feasibility for ecological evaluation.

**Results:**

Structural equation models showed that negative interpretation bias was a direct predictor of worst psychological adjustment (higher depression and anxiety, and lower well-being and resilience; *χ*^2^_9_=7.57; root mean square error of approximation=0.000). Further, rumination mediated the influence of interpretation bias in anxiety (*P*=.045; 95% CI 0.03-3.25) and resilience (*P*=.001; 95% CI −6.34 to −1.65), whereas reappraisal acted as a mediator of the influence of both attention (*P*=.047; 95% CI −38.71 to −0.16) and interpretation biases (*P*=.04; 95% CI −5.25 to −0.12) in well-being.

**Conclusions:**

This research highlights the relevance of individual processes of attention and interpretation during periods of adversity and identifies modifiable protective factors that can be targeted through online interventions.

## Introduction

The occurrence of major stressors (eg, serious illnesses, loss of beloved ones, job loss, and economic difficulties) has a deep psychological impact on individuals in terms of both increased depression and anxiety symptoms [1,2], and reduced well-being [3]. Extant empirically supported “diathesis-stress” models [4] highlight how such a psychological impact would be the result of life stressors, particularly in individuals who have pre-existing vulnerabilities. Among those vulnerabilities, cognitive models have posited the relevant role of individual differences in cognitive processes of attention and interpretation [5,6]. These processes are thought to be on the basis of dysfunctional emotion and stress regulation [7], and are key mechanisms in the onset and maintenance of affective psychopathology in response to stress [5].

Experimental psychopathology research largely supports these assumptions. Stress-related disorders, such as depression and anxiety, have been consistently associated with a marked tendency to process (ie, attend and interpret) emotional information in a negative manner in laboratory studies. For instance, while eye-tracking studies have shown that higher psychological well-being levels are associated with attentional biases toward positive information [8,9], this type of research also shows that depressed individuals are characterized by sustained attention and difficulties disengaging from negative information [8,10], as well as reduced attention toward positive information [11,12]. Furthermore, a biased tendency to interpret ambiguous scenarios in a negative manner has been consistently observed in experimental studies in both depressed [13] and anxious individuals [14].

Conversely, cognitive models posit that attention and interpretation biases would contribute to stress-related psychopathology through their contribution to dysfunctional stress and emotion regulation [15]. This claim has also been experimentally supported. For instance, it has been found that, after negative mood induction, a participant who spent more time attending to positive emotional information (ie, happy faces) recovered faster from induced transient negative moods, whereas sustained attention to negative emotional information predicted impaired stress recovery [16,17]. Importantly, individual differences in the habitual use of emotion regulation strategies are related to the modulation of these forms of affective processing, contributing to maladaptive stress regulation. The habitual use of reappraisal, a strategy typically associated with enhanced stress recovery [18], has been found to modulate attention directed to negative information [19]. Further, the momentary use of reappraisal has been found to predict higher positive interpretation biases to solve ambiguities [20]. In contrast, rumination (ie, passively and repetitively focusing on the symptoms and consequences of distress [21]) hinders the ability to recover from stress [22]. Rumination has been found to interfere with adaptive attention processing, being related to both attention biases toward negative information [23] and negative interpretation biases [24].

In summary, laboratory studies have consistently supported relations between cognitive biases and processes of emotion dysregulation to account for stress-related psychopathology and reduced psychological well-being. Yet, the ecological manifestation of these cognitive biases, as they unfold during the occurrence of real-life major stressors, still remains largely unstudied. This step is crucial to understand how these processes may act as mechanisms of vulnerability and/or resilience to the onset and/or maintenance of psychological impairments in the face of major stressful experiences. This study aimed to provide an initial examination of the interplays among cognitive biases, emotion regulation processes, and outcomes of psychological adaptation to major stress, introducing a novel online method that allows for remote ecological assessment of attention and interpretation biases during daily life functioning. The method was based on a computerized paradigm that allows the online assessment (and intervention) of both attention and interpretation biases during the processing of emotional information [17]. It comprises a modified version of the scrambled sentence task (SST) [25], where participants are asked to create (interpret) self-referent statements using 5 out of 6 presented words (eg, “the future looks very dismal” or “the future looks very bright”) derived from unambiguous items (eg, “looks the future bright very dismal”), where eye tracking–based techniques are used to monitor the time attending to negative and positive information (eg, “dismal” vs “bright”). Using this method to manipulate attention and interpretation biases under experimental conditions, it has been shown that emotional biases in attention and interpretation are causally involved in the spontaneous use of rumination and the ability to use reappraisal in response to laboratory-based negative situations [17]. However, as highlighted above, less is known about the relations between attention and interpretation biases and emotion regulation strategies when people are faced with major stressors. This study integrated an online evaluation of these mechanisms during the occurrence of a global major stressor (the beginning of the COVID-19 pandemic in early 2020) and, specifically, during the restrictive lockdown implemented to face the pandemic at the end of March to the beginning of April of that year.

The COVID-19 pandemic had a dramatic impact on not only public health and socioeconomic status [26] but also citizens’ psychological functioning. It is well-established that pandemic situations are related to increased levels of stress and have a large impact on the prevalence of psychopathologies, such as anxiety and depression, in the general population [27,28]. Until date, the available data with regard to the psychological impact of the COVID-19 pandemic are in this line. Despite the heterogeneity and methodological issues that initial research in the context of urgency had to face [29], extant literature has consistently shown a significant reduction in well-being and an increase in the rates of mental health problems in the general population as a result of the pandemic. For instance, a previous study [30] evaluated a representative sample of 7236 Chinese participants and found a significant increase in the overall prevalence of depressive symptoms (20.1%), anxiety symptoms (35.1%), and poor sleep quality (18.2%) during the beginning of the COVID-19 pandemic. Studies in other geographical areas obtained similar results, reporting increased rates of anxiety and depression in the general population due to the pandemic [28,31]. In Spain, one of the countries more strongly affected by the COVID-19 pandemic during the first half of 2020, studies assessing nationally representative samples found that the rates of clinical depression and anxiety were 22.1% and 19.6%, respectively, in that period [32]. Additionally, these studies found a significant reduction in well-being associated with social (eg, loneliness) and mental health factors (eg, anxiety reactivity) derived as a result of the COVID-19 pandemic. These data highlight the urgent need to understand the underlying factors that have a potential role in reducing the psychological impact of major stressors, such as those derived from the COVID-19 situation.

In the first attempt, some studies assessed self-reported indicators of resilience and their contributions to psychological adjustment during the pandemic. Using equational structural models, it was shown that optimism and positive beliefs about the world might facilitate posttraumatic growth. On the contrary, suspiciousness and intolerance to uncertainty were related to posttraumatic stress symptoms during the pandemic [33]. Further research showed that self-reported positive reappraisal style (ie, the ability to take perspective and to reinterpret situations) was the strongest factor related to the ability to face adversities derived from COVID-19 [34]. Taken together, these findings underlined the relevance of individual differences in cognitive processing (eg, optimism and/or positive beliefs about the world) and adaptive emotion regulation processes (eg, positive reappraisal style) to facilitate psychological adjustment when facing the stress derived from the COVID-19 pandemic. With this study, we aimed to establish whether ecological online assessments of cognitive biases would relate to maladaptive processes of emotion regulation and, ultimately, to psychological adaptation in the face of corona-related major stressors.

To the best of our knowledge, no study has ecologically assessed the relations between these factors and mental health outcomes in the context of major stressors derived from the COVID-19 pandemic. Therefore, the aim of this study was to analyze the daily life role of cognitive biases (i.e., attention and interpretation biases) in emotion regulation and symptom development when facing major stressors. More specifically, the main aim of the study was to test, using structural equation modeling, the predictive role of ecological cognitive biases to emotional information (attention and interpretation biases) and emotion regulation strategies (use of reappraisal and brooding rumination in response to stress during the initial weeks of the pandemic) in psychological maladjustment to major stress (ie, higher depression and anxiety, and lower well-being and resilience). Cognitive biases were monitored through an online test that was completed by participants during the initial weeks of the restrictive lockdown experienced in Spain as a result of the COVID-19 pandemic. In line with previous research supporting the interrelation between cognitive biases and emotion regulation strategies to account for stress regulation, we first hypothesized that cognitive biases (ie, attention and interpretation biases) would have a direct effect on psychological adjustment. Moreover, beside these direct effects, we hypothesized that the use of emotion regulation strategies (ie, rumination or reappraisal) would act as mediators in the pathways between cognitive biases (ie, attention and interpretation biases) and psychological adjustment to stress. We specifically expected that negative cognitive biases would enhance the use of rumination, leading to worse psychological adjustment to stress (ie, higher depression and anxiety, and less well-being and resilience). Conversely, we hypothesized that negative cognitive biases would hinder the use of reappraisal as a strategy to facilitate psychological adjustment (ie, less depression and anxiety, and higher well-being and resilience).

## Methods

### Participants

A total of 100 participants voluntarily completed an online survey regarding their psychological functioning during the COVID-19 lockdown in Spain, during the period between the end of March and the beginning of April 2020 (3/4 weeks following the beginning of a very restrictive lockdown to prevent the expansion of COVID-19 in this country). Immediately after completing the online survey, all participants were invited to complete an online attention and interpretation experimental task through a custom-built Android smartphone app. Twenty participants were excluded owing to technical issues with the online test (10/100, 10% of the sample) or dropouts (10/100, 10% of the sample). Therefore, the final sample with completed measures of attention and interpretation biases during the lockdown included 80 participants (female: 62/80, 78%), and the mean age was 27.7 years (SD 11.3 years). The study was conducted in accordance with the Declaration of Helsinki 2013, and it was approved by the ethical committee of the Faculty of Psychology at the Complutense University of Madrid (reference 2019/20-028).

### General Procedure

Participants were recruited via extensive advertising on social media and social networks. First, all participants completed an online survey administered via Qualtrics Software [35] (see Multimedia Appendix 1 for the Checklist for Reporting Results of Internet E-Surveys [CHERRIES]). This survey comprised an informed consent form, and a series of sociodemographic and self-reported psychological measures (see below). Immediately afterwards, participants were invited (via email) to install and complete on their phones an adaptation of the SST [25] designed for online remote assessment of attention and interpretation biases during daily life functioning. This was done through a novel smartphone app, adapting the computerized version of the SST for online assessment [17].

### Materials

#### Self-reported Measures

Depressive symptoms were assessed through the Center for Epidemiological Studies on Depression-8 scale [36]. Participants reported how often they had experienced depression-related symptomatology during the last week on a 4-point Likert scale (ranging from 0 [none or almost none of the time] to 3 [all or almost all of the time]). Higher values represent the presence of depression symptoms, whereas lower values represent the absence of depressive symptomatology. The reliability in our study was good (α=.84).

Participants’ anxiety symptoms were assessed through the Generalized Anxiety Disorder-7 scale [37]. It has 7 items and uses a 4-point Likert scale (from 0 [not at all sure] to 3 [nearly every day]), where general anxiety-related symptoms (irritability, worry, etc) are assessed with reference to the last 2 weeks. Higher values represent the presence of anxiety symptoms, whereas lower values represent the absence of anxious symptomatology. In this study, the internal consistency was good (α=.85).

Participants’ psychological well-being was assessed using the Warwick-Edinburgh Mental Well-being Scale (WEMWBS) [38]. It has 14 items and uses a 5-point Likert scale (from 1 [none of the time] to 5 [all of the time]) to measure a broad range of factors of psychological well-being, including emotional aspects, cognitive dimensions, interpersonal relationships, and positive functioning. Higher values represent higher levels of well-being. In this study, the internal consistency of the scale was very good (α=.92).

Finally, participants’ resilience was measured using the Brief Resilience Scale [39]. This scale has 6 items and uses a Likert scale ranging from 1 (strongly disagree) to 5 (strongly agree). It conceptualizes resilience as the ability to bounce back from adversity or stress. In this study, questions were framed in relation to specific abilities to deal with the experience of COVID-19 stressors and were framed with reference to the last week (ie, during the lockdown period). Higher values represent a better ability to deal with the situation. In the present sample, its internal consistency was good (α=.82).

The use of emotion regulation strategies during the lockdown was evaluated. The use of rumination as an emotion regulation strategy since the beginning of the COVID lockdown was assessed through the brooding rumination subscale from the Ruminative Response Scale [40]. It comprises 5 items and uses a Likert scale (from 1 [almost never] to 4 [almost always]). Furthermore, the use of reappraisal as an emotion regulation strategy since the beginning of the COVID lockdown was evaluated through the reappraisal subscale from the Emotion Regulation Questionnaire [41]. This scale comprises 4 items and uses a Likert scale (from 1 [totally agree] to 5 [totally disagree]). Higher values represent a marked tendency to use rumination or reappraisal. Both scales showed adequate internal consistency in our study (α=.76 and α=.79, respectively).

To capture the influence of the lockdown situation on participants’ emotion regulation and psychological adjustment, all measures were framed with reference to the 2 weeks before the assessment.

#### Online Attention and Interpretation Bias Task

Attention and interpretation biases were assessed using an online variant of the SST [25], adapted from the computerized procedure for online attention and interpretation bias assessment that has been previously validated [42]. A total of 15 scrambled sentences with 6 words (eg, “looks the future bright very dismal”) were presented to the participants. The number of trials was established based on previous extensive piloting of sufficient required SST trials to obtain reliable cognitive bias indices related to stress vulnerability and depression status (Martín-Romero, unpublished data, July 2021). Participants were instructed to mentally unscramble the sentences, as fast as possible, using only 5 out of the 6 words, to create a grammatically correct and meaningful sentence. These sentences could only be unscrambled with a negative or a positive meaning (eg, “the future looks very dismal” or “the future looks very bright”). Participants were instructed to unscramble the words into the valid sentence that first came to their mind. To control for the influence of word positioning, emotional words (ie, positive or negative) were always displayed in the second and fifth positions. Additionally, these positions were counterbalanced, with positive and negative words similarly allocated in the second and fifth positions across trials.

**Figure 1:**
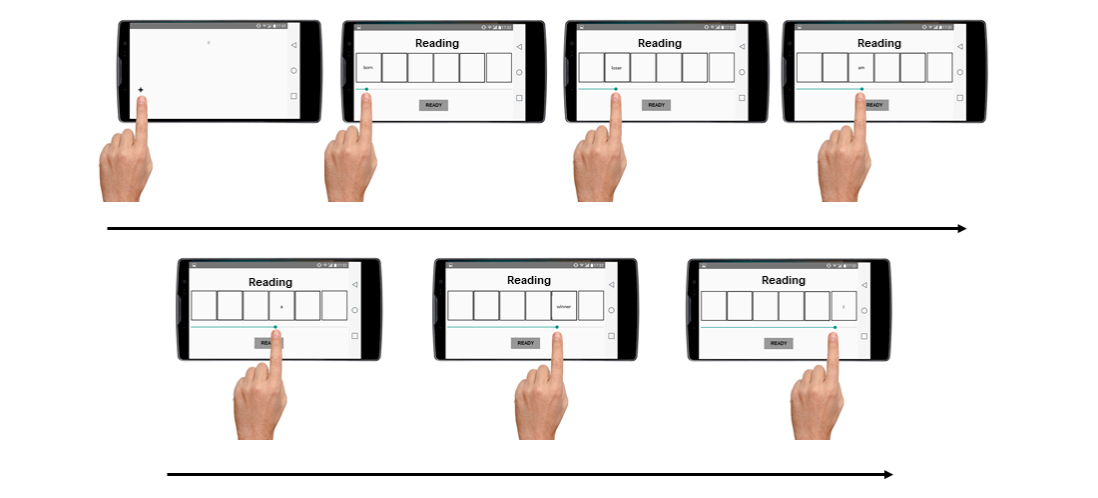
Example of the reading phase.

The task was completed on participants’ smartphones. Each trial started with a fixation cross in the left position of the screen to promote natural left-to-right reading patterns. Participants were asked to press the cross with their finger to start the trial. Immediately after, a reading phase started, where participants had to read and mentally unscramble the words in a limited time of 14 seconds. Using a moving window procedure, the 6 words were hidden in individual boxes. In order to read them, participants had to move their finger throughout a scroll bar below the boxes to unhide the corresponding word. Once participants moved their finger from one word to another, the previous words were hidden again. During this reading phase, the position of the finger on the screen was monitored, allowing to compute the time spent (in milliseconds) reading (attending to) each word of the scrambled sentence, and thus, the proportion of total time reading negative over positive words could be assessed (ie, negative attention bias). After the time limit, or when participants decided (pressing a “Ready” button), the final response phase began (Figure 1).

**Figure 2:**
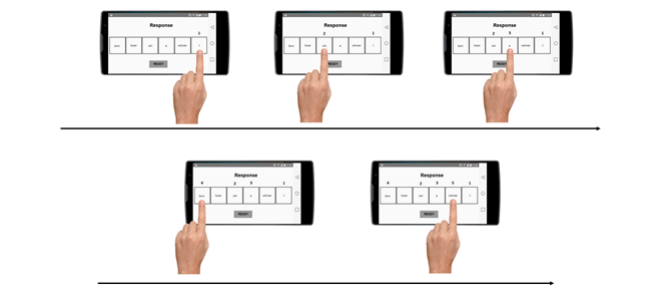
Example of the response phase.

In the response phase, all words were unhidden. With a time limit of 7 seconds, participants had to create a meaningful sentence by pressing, as fast as possible and in the appropriate order, the corresponding chosen series of 5 words. If participants made any mistake during the construction of the sentence, they could modify it by unselecting the wrong word and selecting a new one (Figure 2). Once the 5 words were selected, participants pressed the “Ready” button at the bottom of the screen and started a new trial. The system recorded responses for each trial to compute the interpretation bias index (see below).

#### Attention and Interpretation Bias Indices

The task was designed to allow for online assessment of the total time attending to negative over positive words during the reading phase, as well as the proportion of negative over positive interpretations made during the response phase. The program registered the total time (in milliseconds) that participants spent reading (attending to) negative and positive words. We analyzed the reliability of each measure. Reliability analysis showed very good reliability for both measures of total time attending to positive (α=.87) and total time attending to negative (α=.90) stimuli. Following previous studies [42], an attention bias index was computed by dividing the total time attending to negative words by the total time attending to both emotional (ie, positive and negative) words. Values above 0.5 are indicative of an attention bias toward negative information, whereas values below 0.5 are indicative of an attention bias toward positive information. The program also computed the number of positive and negative grammatically correct sentences that were unscrambled by each participant during the response phase. An interpretation bias index was computed by dividing the number of negative sentences by the total number of unscrambled sentences (ie, positive and negative). Split-half reliability analysis showed good reliability for this index (*r*=0.75; ρ=0.86). As with the attention bias index, values above 0.5 indicate a negative interpretation bias, whereas values below 0.5 indicate a positive interpretation bias.

### Data Analysis Plan

Once we established the good psychometric properties of the cognitive bias measures, in terms of their reliability for ecological online attention and interpretation bias indexing, we conducted the main analyses in the study.

#### Demographics, COVID-19–Related Variables, and Psychological Measures

We conducted descriptive analyses of demographics and psychological measures of participants, including gender, age, civil status, and education level, as well as computed the mean (SD) levels of self-report measures and online cognitive bias assessments.

#### Relations Between Cognitive Biases and Emotion Regulation Strategies With Psychological Adjustment Indices

We conducted a series of Pearson bivariate correlations to analyze the relations between the attention and interpretation biases and emotion regulation strategy measures with psychological adjustment indices.

#### Structural Equation Models

We tested an equation structure model including those variables that were significantly correlated. Thus, we tested a model where attention and interpretation biases act as exogenous variables, all of which predicted psychological adjustment (ie, depression, anxiety, well-being, and resilience) directly and also indirectly through the use of emotion regulation strategies (ie, use of rumination or reappraisal), which would act as mediators. Moreover, we tested the reverse model where psychological adjustment variables were introduced as predictors, emotion regulation strategies as mediators, and cognitive bias indexes as outcome variables. The estimation of the standardized parameters of the model followed the full information maximum likelihood (FIML) estimation method. To test the adjustment of our model, we used the following standard criteria [43]: (1) χ^2^, a nonsignificant value indicates a perfect fit; (2) χ^2^/df, a value lower than 2 indicates a good fit; (3) comparative fit index and Tucker-Lewis index, a value ≥0.95 indicates a good fit; (4) root mean square error of approximation, a value ≤0.05 indicates a good fit; (5) standardized root mean square, a smaller value indicates a better fit between the observed data and the tested model; and (6) Akaike information criterion, a lower value indicates the preference for selecting a model when compared to another model. Moreover, we used the Mardia coefficient for assessing multivariate normality (a value ≤5 indicates the possibility to assume multivariate normality) [44]. Finally, the hypothesized mediation pathways within the model (ie, cognitive bias → emotion regulation strategy → psychological adjustment outcome) were tested via the estimation of indirect effects within the final model. All the structural equation models were tested using AMOS v18.0 (SPSS Inc). A *P* value <.05 was used to determine statistical significance in all analyses.

## Results

### Demographics, COVID-19–Related Variables, and Psychological Measures

Descriptive data of demographics and psychological measures of the participants in this study are shown in Table 1.

**Table 1:** Descriptive data of demographics and psychological measures.

Variable		Value (N=80)
Gender: female, n (%)		62 (78)
Age (years), mean (SD)		27.7 (11.3)
**Civil status, n (%)**		
	Single	34 (43)
	Married	34 (43)
	In a relationship	7 (9)
	Divorced/widower	5 (6)
**Educational level, n (%)**		
	Without studies	0 (0)
	Primary school	0 (0)
	High school	43 (54)
	University graduate	37 (46)
Negative interpretation bias, mean (SD)		0.28 (0.23)
Negative attention bias, mean (SD)		0.51 (0.03)
Rumination level, mean (SD)		11.54 (3.63)
Reappraisal level, mean (SD)		12.45 (3.37)
Depression level, mean (SD)		7.06 (2.81)
Anxiety level, mean (SD)		2.60 (3.04)
Well-being level, mean (SD)		48.25 (8.08)
Resilience level, mean (SD)		18.44 (4.65)

### Relations Between Cognitive Biases and Emotion Regulation Strategies With Psychological Adjustment Indices

Depression and anxiety were significantly positively related to the use of rumination (*r*=0.398 and *r*=0.450, respectively) and negative interpretation biases (*r*=0.619 and *r*=0.488, respectively) during the lockdown. Resilience and well-being were also significantly but negatively related to the use of rumination (*r*=−0.575 and *r*=−0.502, respectively) and negative interpretation biases (*r*=−0.536 and *r*=−0.574, respectively) during the lockdown, and significantly positively related to the use of reappraisal during the lockdown (*r*=0.374 and *r*=0.330, respectively). Moreover, all these psychological adjustment variables (ie, levels of depression, anxiety, resilience, and well-being) were significantly related among each other (all *P*<.001).

With regard to cognitive biases and the use of emotion regulation strategies during the lockdown, higher use of rumination was significantly associated with lower use of reappraisal (*r*=−0.293) and with higher levels of negative interpretation biases (*r*=0.543). In the case of the use of reappraisal, it was negatively related to both negative attention and interpretation biases (*r*=−0.224 and *r*=−0.275, respectively) (see Multimedia Appendix 2 for all correlation results).

### Structural Equation Models

The Mardia coefficient yielded a value of 2.15, which is far below the critical value (±5), assuming multivariate normality in our data [44]. Based on the previous bivariate correlation analysis and following the predictions from current cognitive models [15,45], we tested an equation model where psychological adjustment variables (ie, depression, anxiety, resilience, and well-being) were predicted by cognitive biases directly and/or indirectly through the use of emotion regulation strategies. All the goodness-of-fit indices are shown in Table 2.

**Table 2:** Goodness-of-fit indices for the tested models.

Model	Chi-square (*df*)	*P* value	*χ*^2^/*df*	CFI^a^	TLI^b^	RMSEA^c^ (90% CI)	SRMR^d^	AIC^e^
Model 1^f^	62.4 (15)	<.001	4.16	0.79	0.61	0.20 (0.14-0.25)	0.1061	120.1
Model 2^g^	137.2 (15)	<.001	9.14	0.46	−0.01	0.321 (0.27-0.37)	0.2878	195.1
Model 1R^h^	7.6 (9)	.58	0.84	1	1.02	0.000 (0.00-0.11)	0.0554	77.56

^a^CFI: comparative fit index.

^b^TLI: Tucker-Lewis index.

^c^RMSEA: root mean square error of approximation.

^d^SRMR*:* standardized root mean square.

^e^AIC: Akaike information criterion.

^f^Model 1: initial model.

^g^Model 2: alternative model.

^h^Model 1R: initial model respecified.

**Figure 3:**
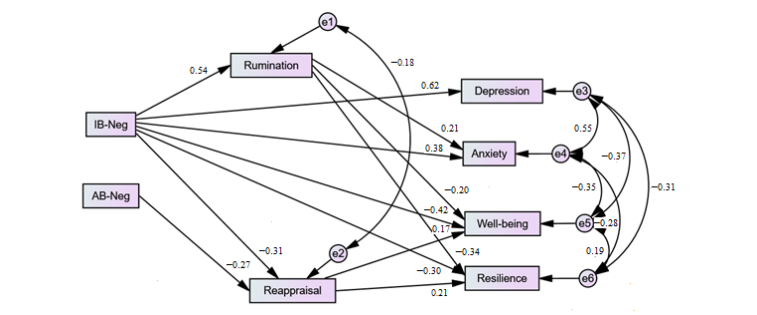
The respecified model (Model 1R) with standardized regression weights. AB-Neg: negative attention bias; IB-Neg: negative interpretation bias.

As shown in Table 2, the goodness-of-fit indices were better for our hypothesized model (Model 1) than for the alternative reverse model (Model 2). However, since the fit of our initial model (Model 1) was poor, respecification was carried out following Wald and Lagrange multiplier tests [46]. All paths with nonsignificant *P* values were removed consecutively. Only the path *rumination* to *depression* was removed. No additional paths were included in the model (Figure 3). The final respecified model (Model 1R) showed very good fit in all of the indices (Table 2).

Finally, indirect effects were tested using a bias-corrected bootstrap estimation (2000 bootstrap samples with 95% CI). As shown in Table 3, significant indirect effects were found between negative interpretation biases and anxiety and resilience via rumination (*P*=.045 and *P*=.001, respectively). Additionally, via the reappraisal path, indirect effects between negative cognitive biases (ie, both attention and interpretation biases) and well-being were statistically significant (*P*=.047 and *P*=.04, respectively).

**Table 3:** Bootstrap mediational analysis.

Variable		Indirect effects (95% CI)		SE	*P* value
		Lower	Upper		
**Indirect effect via rumination**					
	Interpretation bias → anxiety	0.032	3.246	0.819	.045
	Interpretation bias → well-being	−8.736	0.492	2.344	.08
	Interpretation bias → resilience	−6.341	−1.647	1.192	.001
**Indirect effect via reappraisal**					
	Interpretation bias → well-being	−5.251	−0.117	1.226	.04
	Interpretation bias → resilience	−3.792	0.079	0.981	.08
	Attention bias → well-being	−38.714	−0.159	8.904	.047
	Attention bias → resilience	−24.647	0.411	6.241	.07

## Discussion

The main aim of this study was to assess the predictive role of cognitive biases and emotion regulation strategies on different indices of psychological adjustment to a major stressor, the COVID-19 lockdown (namely, lower depression and anxiety, and higher well-being and resilience in the face of experienced corona stress). Using structural equation modeling, we analyzed how ecological online assessments of cognitive biases (ie, attention and interpretation biases remotely measured through a novel app-based system integrating the SST) were directly related to the outcomes of psychological adjustment to corona stress and/or indirectly related to them through the use of emotion regulation strategies during the lockdown (ie, rumination and reappraisal in response to experienced negative events). Our results highlight the central role of negative interpretation bias as a vulnerability factor during the lockdown period, accounting for significant variance in all indicators of psychological adjustment to major stress (namely, depression and anxiety, and well-being and resilience in the face of experienced corona stress), above and beyond attention bias and the use of emotion regulation strategies (ie, rumination and reappraisal). We also found mediation effects of the use of strategies (rumination and reappraisal) between negative cognitive biases and psychological adjustment outcomes.

These results show the direct effect of cognitive biases on psychological adjustment to the COVID-19 lockdown, and support our initial hypotheses. It is worth noting that the sentences used in our online SST paradigm (remotely measured through a novel app-based system) were related to different central cognitive schemas for psychopathology and well-being (such as self-concept, world, and future beliefs). It seems that the interpretation of those sentences in a negative manner (eg, “the future looks very dismal”), in contrast to the interpretation in a positive manner (eg, “the future looks very bright”), emerged as an important risk factor that enhanced the impact of the major stressful situation on psychological functions (ie, increasing depression and anxiety levels) and reduced positive functioning variables, such as psychological well-being and resilient responses to corona stress. This result is consistent with previous research showing that individual differences in positive beliefs about the world were one of the major predictors of posttraumatic growth in the face of corona stress [32]. In contrast, attention biases to negative versus positive information did not have any direct effect on psychological adjustment. These findings suggest that the role of biased attention as a direct correlate of psychological functioning (ie, depression and anxiety levels or well-being and resilience) may be limited. Multiple studies support the idea that attention biases might exert indirect influences in psychological functioning through their influence on elaborative processes such as interpretation bias [47-49]. However, we did not find any statistical relation between attention and interpretation biases. Therefore, it is plausible that, despite the high reliability of the attention bias index, the task used for assessment was not able to fully capture the actual attentional processes in the present sample. Moreover, it might be plausible that the negative interpretation bias index introduced in our model accounted for all the variance in psychological outcomes explained by the negative attention bias index. In fact, previous research has also shown that attention bias indices did not demonstrate significant relevance to directly account for psychological symptoms when other related elaborative cognitive processes, such as memory biases, were modeled together [50]. Thus, the results found regarding attention biases in this study should be considered cautiously.

As previously mentioned, we also analyzed the mediational role that the use of emotion regulation strategies during the COVID-19 lockdown played in the interplay between negative cognitive biases and consequent psychological adjustment outcomes. Analysis showed that the use of rumination emerged as a significant mediator between interpretation bias and anxiety and resilience. Regarding the use of reappraisal, our results showed that while reappraisal partially mediated the relation between negative interpretation bias and well-being, it totally mediated the association of negative attention bias with well-being. These findings are in line with current theories with regard to the major role that cognitive processes play on emotion regulation [15,45]. Our findings indicate the relevance of interpretation biases as a particularly central mechanism to hinder or buffer the impact of adverse situations, such as those derived from the COVID-19 emergency and the resulting lockdown period during March/April 2020. This is in line with former empirical evidence in the context of COVID-19. For instance, a positive appraisal style was found to be the major contributor for resilience during the COVID-19 pandemic [34]. However, our data go beyond previous studies using self-reported measures of these processes and suggest that direct ecological assessments of negative interpretation biases, as they manifest during daily functioning, might reduce the ability to use positive reappraisal, hindering psychological adjustment.

Taken together, our results support the idea that individual differences in the way reality is perceived and interpreted (ie, the construction of self-relevant meanings from ongoing experiences) may be central to increase (or reduce) the psychological impact of ongoing adversities (such as the one experienced during the COVID-19 pandemic). In times of major stress and uncertainty, as the period under study, difficulties in accessing standard in-person resources of psychological assistance may emerge. Conversely, applied work to intervene in these biases could be efficiently integrated into remote online interventions. This includes novel online protocols to directly train positive interpretation biases, with consistent results for changes in emotion regulation and clinical outcomes [51], as well as cognitive tools designed to actively train attention operations involved in interpretation bias change and adaptive emotion regulation [42]. Therefore, future research is warranted to adapt these promising tools for easy access online implementations that can facilitate stress regulation in daily life and positive psychological functioning during the occurrence of major adversities.

It is worth noting the strengths and limitations of this study. As for the strengths of the study, to our knowledge, this is the first study that has ecologically assessed cognitive biases of affective processing during the occurrence of a major stressor, such as the COVID-19 lockdown of early 2020. Furthermore, the adaptation of a previously validated paradigm to remotely assess attention and interpretation biases [42] increases the ecological validity of the present results in terms of the indices of attention and interpretation bias performance. Furthermore, the reliabilities of these cognitive bias measures were very good, supporting their feasibility for use in online remote assessments during the occurrence of major stressors. Moreover, the study was conducted in Spain, which was one of the countries more dramatically hit by the COVID-19 situation at the time of the study, with data being collected during a very restrictive lockdown. Given all these conditions, we were able to test purported mechanisms of psychological (mal)adjustment to major stress with considerable ecological validity.

With regard to limitations, our sample was relatively small. Yet, our current findings were consistent across different forms of psychological adjustment to major stress. This supports the relevance of these findings and informs about the potential of further investigating these models in more representative samples to fully determine the role of cognitive affective processes in buffering the impact of major stressors. Furthermore, the mobile app developed to remotely assess cognitive biases could only be adapted to work on Android smartphones, limiting the number of screened participants that could be included in the study, and thus, partially restricting the representativeness of the sample. Future research is warranted to adapt this new tool for other operating systems, which will allow access to bigger samples and to replicate these initial findings in representative samples under different related conditions of major stress and adversity. Furthermore, cognitive theories have pointed out that attention and interpretation biases interplay with other cognitive biases such as memory biases [47]. However, in this study, only attention and interpretation were assessed. Future studies should also consider developing ecological online assessments of memory biases to analyze their specific roles in accounting for emotion regulation and psychological functioning when facing major stressors in daily life.

In summary, our study presents a novel approach that allows the analysis of the interplay of cognitive biases, emotion regulation strategies, and psychological adjustment when facing major stressors, which are assessed in naturalistic settings.

## Appendix

Multimedia Appendix 1 Checklist for Reporting Results of Internet E-Surveys (CHERRIES).

Multimedia Appendix 2 Bivariate correlations among cognitive biases, use of emotion regulation strategies, and psychological variables (depression, anxiety, resilience, and well-being).

## Abbreviations

SST: Scrambled Sentence Task
