# Supplementary material for: An Online Assessment to Evaluate the Role of Cognitive Biases and Emotion Regulation Strategies for Mental Health During the COVID-19 Lockdown of 2020: Structural Equation Modeling Study
Source: JMIR Ment Health. 2021 Nov 2;8(11):e30961. doi: 10.2196/30961 (PMC8565804; doi:10.2196/30961)
Supplement: Multimedia Appendix 1 [file mental_v8i11e30961_app1.docx]

# Appendix 1. Checklist for Reporting Results of Internet E-Surveys (CHERRIES).

| **Item Category** | **Checklist Item** | **Explanation** |
| --- | --- | --- |
| **Design** | |  |
|  | Describe survey design | Participants were recruited from the general population thought extensive advertising in social media and social networks. Only adults (>18) were recruited.  They completed an online survey in the web page created for the study. After that, they were contacted via email to complete an online task though a custom-built Android smartphone App. |
| **IRB** | |  |
|  | IRB approval | The study was conducted in accordance with the Declaration of Helsinki 2013 and it was approved by the ethical committee of the Faculty of Psychology at the Complutense University of Madrid (ref. 2019/20-028). |
|  | Informed consent | Information of the aim of the study, study design (duration of the study, number and duration of questionnaires) and data storage was presented in the web page of the study. All the answers were stored in Qualtrics website and Google Firebase. Also, in the first page of the online survey, participants were informed about their voluntarily participation and data protection, and declared to consent before completing the survey. |
|  | Data protection | Only researchers had access to Qualtrics website or Google Firebase where survey and task responses were stored. Participants registered those responses by introducing their email address after completing the informed consent. In the case of the App, they created an account with their email and a password. |
| **Development and pretesting** | |  |
|  | Development and testing | The online platform, survey items and the tasks were pre-tested in a study with students of the Complutense University of Madrid and Autonoma University of Madrid. The online system was rated as highly usable and participants found the system as highly useful and satisfying. |
| **Recruitment process and description of the sample having access to the questionnaire** | | |
|  | Open survey versus closed survey | The survey was open but the Android App was a closed survey. |
|  | Contact mode | Initial contact was made though internet, as information about the study directed potential participants to complete the open survey though the webpage. After that, participants were contacted via email to complete the online task though a custom-built Android smartphone App. |
|  | Advertising the survey | Announcement of the survey was published in the research group webpage as well as published in social media. Information about the aim of the study, and the research group web page was included. |
| **Survey administration** | |  |
|  | Web/E-mail | Qualtrics and Android smartphone App. |
|  | Context | The web site in which the survey was posted described the aim of the study and introductory information about psychological processes such as emotion regulation during Covid-19 lockdown in Spain.  The content of the website could have attracted to participants interested in psychological processes related to the situation of Covid-19, as well as could have been especially attractive for students of psychology. |
|  | Mandatory/voluntary | Completing the online survey was voluntary after entering in the website. |
|  | Incentives | No incentives were offered to participants. |
|  | Time/Date | Data was collected during the end of March and the beginning of April 2020 (three/four weeks following the beginning of very restrictive lockdown to prevent the expansion of COVID-19 in Spain) |
|  | Randomization of items or questionnaires | Items were not alternated or randomized. |
|  | Adaptive questioning | Items from the baseline questionnaire were conditionally displayed for demographic information (ie, educational level) |
|  | Number of Items | Each page of the survey presented a single questionnaire. Therefore, items in each page differed, depending on the number items that each questionnaire contained. |
|  | Number of screens (pages) | As we included 7 questionnaires in the survey, it contained 7 pages. |
|  | Completeness check | Responding to all items was required. In case of incomplete answers, the system showed a message “please, respond to all questions” before the participation could continue. Only in Items of demographic information a non-response option such as “rather not say” was included. |
|  | Review step | Before sending the answers, participants were able to access to previous pages of the questionnaire though a Back button to review or change answers. |
| **Response rates** | |  |
|  | Unique site visitor | Views of the website were not registered.  Participants completed the online survey and after that, all participants were invited to complete an online attention and interpretation experimental task through a custom-built Android smartphone App.  Only participants who completed the entire questionnaire and experimental task were included in the analysis. |
|  | View rate | As views of the website were not registered, calculation of views rated is not possible. |
|  | Participation rate | Only responses were registered by the website when completing the first page of the survey. |
|  | Completion rate | 0.8 |
| **Preventing multiple entries from the same individual** | |  |
|  | Cookies used | The system assigned an identifier to each participant´s device. Duplicated entries were eliminated before analyses, preserving the one entirely completed. |
|  | IP check | IP address was not used to identify potential duplicate entries from the same user. |
|  | Log file analysis | Multiple entries were identified by the registration of the email address of participants. Duplicated entries were eliminated before analyses, preserving the one entirely completed. |
|  | Registration | Multiple entries in the Android app were not registered as the system prevented participants to register the response twice. |
| **Analysis** | |  |
|  | Handling of incomplete questionnaires | Only completed questionnaires were analyzed. |
|  | Questionnaires submitted with an atypical timestamp | Time participants took to respond to the questionnaire was not considered. |
|  | Statistical correction | No methods of statistical correction have been applied. |
