# Supplementary material for: An Online Assessment to Evaluate the Role of Cognitive Biases and Emotion Regulation Strategies for Mental Health During the COVID-19 Lockdown of 2020: Structural Equation Modeling Study
Source: JMIR Ment Health. 2021 Nov 2;8(11):e30961. doi: 10.2196/30961 (PMC8565804; doi:10.2196/30961)
Supplement: Multimedia Appendix 2 [file mental_v8i11e30961_app2.docx]

# Appendix 2. Bivariate correlations among cognitive biases, use of emotion regulation strategies, and psychological variables (depression, anxiety, resilience, and well-being).

| Measure  *r* (*P* value) | 1 | 2 | 3 | 4 | 5 | 6 | 7 | 8 | |
| --- | --- | --- | --- | --- | --- | --- | --- | --- | --- |
| 1. Attention bias | 1 |  |  |  |  |  |  |  | |
| 2. Interpretation bias | -0.16(.17) | 1 |  |  |  |  |  |  | |
| 3. Reappraisal | -0.22 (.045) | -0.28(.01) | 1 |  |  |  |  |  | |
| 4. Rumination | -0.08(.51) | 0.54(<.001) | -0.29 (.008) | 1 |  |  |  |  | |
| 5. Depression | -0.11(.34) | 0.62(<.001) | -0.21 (.06) | 0.40 (<.001) | 1 |  |  |  | |
| 6. Anxiety | -0.12(.30) | 0.49(<.001) | -0.03 (.76) | 0.45 (<.001) | 0.68 (<.001) | 1 |  | |  |
| 7. Resilience | -0.002(.99) | -0.54(<.001) | 0.37 (.001) | -0.58(<.001) | -0.55 (<.001) | -0.49(<.001) | 1 |  | |
| 8. Well-being | 0.08(.48) | -0.57(<.001) | 0.33 (.003) | -0.50(<.001) | -0.60 (<.001) | -0.54(<.001) | 0.52 (<.001) | 1 | |
